# The Positive Relationship between Moderate-to-Vigorous Physical Activity and Bone Mineral Content Is Not Mediated by Free Leptin Index in Prepubertal Children: The PANIC Study

**DOI:** 10.3390/ijerph18105365

**Published:** 2021-05-18

**Authors:** Annie M. Constable, Josie E. Porter, Danielle Benger, Dimitris Vlachopoulos, Alan R. Barker, Sarah A. Moore, Sonja Soininen, Eero A. Haapala, Kate Westgate, Soren Brage, Ricardo R. Agostinete, Romulo A. Fernandes, Timo A. Lakka

**Affiliations:** 1 Children’s Health and Exercise Research Centre, Sport and Health Sciences, University of Exeter, Exeter EX1 1TX, UK; jp795@exeter.ac.uk (J.E.P.); d.benger@exeter.ac.uk (D.B.); D.Vlachopoulos@exeter.ac.uk (D.V.); A.R.Barker@exeter.ac.uk (A.R.B.); 2 Institute of Biomedicine, Kuopio Campus, University of Eastern Finland, FI-70211 Kuopio, Finland; sonja.soininen@uef.fi (S.S.); Eero.a.haapala@jyu.fi (E.A.H.); timo.lakka@uef.fi (T.A.L.); 3 School of Health and Human Performance, Dalhousie University, Halifax, NS B3H 4R2, Canada; sarah.moore@dal.ca; 4 Social and Health Center, 78300 City of Varkaus, Finland; 5 Faculty of Sport and Health Sciences, University of Jyväskylä, 40014 Jyväskylä, Finland; 6 MRC Epidemiology Unit, University of Cambridge School of Clinical Medicine, Cambridge CB2 0SL, UK; kate.westgate@mrc-epid.cam.ac.uk (K.W.); soren.brage@mrc-epid.cam.ac.uk (S.B.); 7 Laboratory of InVestigation in Exercise (LIVE), Department of Physical Education, Sao Paulo State University (UNESP), Presidente Prudente 19060-900, Brazil; ricardo.agostinete@unesp.br (R.R.A.); romulo.a.fernandes@unesp.br (R.A.F.); 8 Department of Clinical Physiology and Nuclear Medicine, Kuopio University Hospital, University of Eastern Finland, 70210 Kuopio, Finland; 9 Foundation for Research in Health Exercise and Nutrition, Kuopio Research Institute of Exercise Medicine, 70100 Kuopio, Finland

**Keywords:** prepubertal age, free leptin index, accelerometery, bone mass, childhood, DXA, growth

## Abstract

Purpose: Moderate-to-vigorous physical activity (MVPA) positively influences bone mineral content (BMC) in prepubertal children, but it is unknown whether this relationship is partially mediated by free leptin index. The aim of this study was to examine whether the relationship between MVPA and total body less head (TBLH) BMC is mediated or moderated by free leptin index in prepubertal children. Methods: We performed a cross-sectional analysis on 401 children (194 girls) from baseline examinations of the Physical Activity and Nutrition in Childhood Study. We applied the four-way decomposition mediation analysis method to assess whether free leptin index, measured from fasted blood samples, mediated the relationship between accelerometer-measured MVPA and TBLH BMC measured by dual-energy X-ray absorptiometry. Results: MVPA had a positive controlled direct effect on TBLH BMC in girls and boys (β = 0.010 to 0.011, *p* < 0.05). There was no mediation or interaction between MVPA, free leptin index and TBLH BMC in girls or boys (β = −0.000 to 0.001, *p* > 0.05). Conclusion: Our study indicates that MVPA positively influences TBLH BMC through pathways not related to free leptin index in predominantly normal-weight prepubertal children, likely primarily through mechanical loading. The relationships between MVPA, free leptin index and TBLH BMC may be influenced by other factors such as pubertal status and adiposity, so it is unknown whether these observations extend to overweight and obese children at different stages of puberty.

## 1. Introduction

Physical activity (PA) positively influences bone mass through increasing mechanical load and muscle contraction on bones, with evidence indicating that the skeleton is particularly sensitive to mechanical stimuli during pre- and early puberty [1,2,3]. We have previously demonstrated a positive association with moderate-to-vigorous PA (MVPA) in prepubertal children aged 6–9 years [4]. In addition to increasing bone mass biomechanically, PA also has an influence on endocrine factors, such as leptin, which may subsequently affect bone mass [5,6]. Given the importance of bone mass accrual in childhood in protecting against fractures during childhood and adolescence and in determining peak bone mass, exploring the potential mechanisms through which PA may influence bones is important in developing strategies to promote skeletal health [7].

In addition to bone tissue, PA may influence other tissues and hormones, such as leptin. This effect can be influenced by altering energy balance and insulin sensitivity, through changes in lipid metabolism, and through other unknown factors [6,8]. Leptin is highly correlated with adiposity, and we have previously shown that it is important to control for fat mass when investigating the associations between leptin and bone [9]. In children and adolescents aged 8–18 years, PA was negatively associated with plasma leptin, independently of fat mass [10], though in prepubertal children aged 7, there was no association between plasma leptin and moderate-to-vigorous PA (MVPA) or total PA [11]. This may be due to differences in the pubertal status of the participants, which could influence the relationship between PA and leptin. However, it is unclear whether leptin plays a mediatory role in the relationship between PA and bone. In children aged 7–8 years, plasma leptin was not associated with total body less head bone mineral content (TBLH BMC) accounting for fat mass [12], and we have previously shown that the free leptin index was not associated with TBLH areal bone mineral density (aBMD) in children aged 6–9 years independently of fat mass [9]. However, in boys aged 12–14 years, MVPA was negatively associated with plasma leptin, and plasma leptin had an inverse relationship with changes in bone mineral content (BMC) over 2 years [5]. Given the associations between PA and leptin, and the potential influence of leptin on bone, it is possible that leptin may mediate or moderate the relationship between PA and BMC.

The four-way decomposition method of mediation and interaction analysis allows for the total effect (TE) of an exposure on an outcome to be decomposed into a controlled direct effect (CDE), a reference interaction (INTref), a mediated interaction (INTmed), and a pure indirect effect (PIE) [13]. Though previous research has considered relationships between PA, leptin and bone [5], the four-way decomposition approach allows us to explore whether leptin may mediate or moderate the association between PA and BMC. The aim of this study was to apply the four-way decomposition method to examine whether the relationship between MVPA and TBLH BMC is mediated or moderated by free leptin index in prepubertal children aged 6–9 years.

## 2. Materials and Methods

### 2.1. Study Design and Protocol

This study utilized cross-sectional data from baseline of the Physical Activity and Nutrition in Children (PANIC) Study. The PANIC Study is an ongoing longitudinal study in a population sample of Finnish children, with an 8-year controlled lifestyle intervention (ClinicalTrials.gov registration number NCT01803776). Children aged 6–8 years who were registered in the first grade at public school in Kuopio, Finland, were invited to participate in baseline examinations. Of the 736 children who were invited to participate, 512 children (70%) enrolled for baseline investigations in 2007–2009. The study was conducted in accordance with the ethical guidelines of the Declaration of Helsinki and approved by the Research Ethics Committee of the Hospital District of Northern Savo. The parents or caregivers of the children provided their written informed consent, and the children provided their assent to participation.

**Figure 1:**
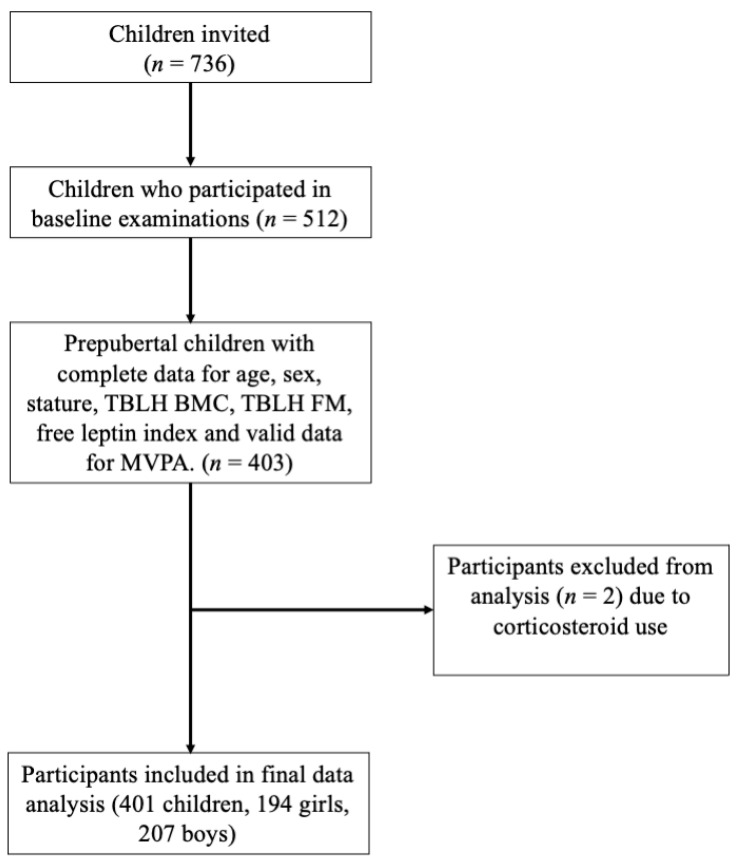
Participant flow chart. Total body less head bone mineral content, TBLH BMC; total body less head fat mass, TBLH FM; moderate-to-vigorous physical activity, MVPA.

For the present analysis, we included prepubertal children with complete data for sex, age, body height, TBLH BMC, TBLH fat mass, and free leptin index, and with valid PA data. We excluded children who were currently taking or had previously taken oral corticosteroids, as this could independently influence BMC [2]. In total, 401 prepubertal children aged 6–9 years (194 girls, 207 boys) were included in the present analysis. The inclusion and exclusion criteria are outlined in Figure 1.

### 2.2. Assessment of General Health and Pubertal Status

General health was assessed using a questionnaire completed by parents or caregivers containing questions regarding regular prescription medication and any diagnosis of chronic diseases or allergies established by the child’s physician. A research physician classified the girls as having entered clinical puberty if their breast development had started and boys if their testicular volume assessed by an orchidometer was ≥4 mL [14].

### 2.3. Anthropometry

Body height was measured three times by a research nurse using a wall-mounted stadiometer to an accuracy of 0.1 cm with children positioned standing in the Frankfurt plane without shoes. Body weight was measured twice by a research nurse using the InBody 720 bioelectrical impedance device (Biospace, Seoul, Korea) to an accuracy of 0.1 kg, with children in a fasted state, having emptied their bladder and standing in light underwear. For body height and body weight, the mean of the values was used in the analyses. Anthropometric measurements were conducted following the standard protocol used in Finnish healthcare, outlined by Saari et al. [15]. Body mass index (BMI) (kg/m^2^) was calculated, and the BMI cut-offs of the International Obesity Task Force (IOTF) were applied to classify children as normal weight, overweight, or obese [16].

### 2.4. Assessment of Bone Mineral Content and Body Composition

TBLH fat mass (kg) and bone mineral content (BMC) (kg) were assessed by a trained and experienced research nurse using the Lunar Prodigy Advance^®^ dual-energy X-ray absorptiometry (DXA) device (GE Medical Systems, Madison, WI, USA) and analyzed using Encore^®^ software, Version 10.51.006 (GE Company, Madison, WI, USA). These measurements were made in accordance with the instructions outlined by manufacturers using standardized protocols. The same DXA device and software were used for all measurements. DXA provides valid and reliable data on BMC and body composition in children (coefficient of variation = 0.01–4.37%) [17,18]. A quality assurance test was performed daily prior to patient measurements. The inspection ensured the functionality and accuracy of the measuring device and method, and tested the setting of the high voltage, the longitudinal and transverse movement of the imaging arc, the shutter mechanism of the beams and the accuracy of the detector. The inspection was performed using a recording table and a standard. A standard is a piece corresponding to a tissue material. Furthermore, for device repeatability monitoring, a weekly testing was performed. According to the quality assurance program, the aluminum phantom was measured once a week. An acceptable measurement result in the repeatability monitoring must not deviate by more than 2% from the long-term average. The effective dose as assessed by whole body scans was 1 to 3 mSv. The primary outcome variable was TBLH BMC, as evidence indicates that for pre- and early pubertal children, BMC is a more accurate and reliable measure than aBMD [19,20,21].

### 2.5. Assessment of Physical Activity

PA was assessed using an individually calibrated combined heart rate and movement sensor (Actiheart, CamNtech Ltd., Papworth, UK) [22,23]. The device was attached to the chest with standard electrocardiogram electrodes (Bio Protech Inc., Wonju, Korea) and set to record heart rate and body movement in 60 s epochs. Participants were instructed to wear the monitor continuously for a minimum of four consecutive days, although some children wore the monitor for up to nine days. As PA patterns differ between weekdays and weekends, the wear period was scheduled to include an entire weekend [24].

Heart rate data were pre-processed using robust Gaussian Process regression [25] and individually calibrated to PA energy expenditure using a maximal exercise test on a cycle ergometer [26]. Intensity was modeled from the combined sensing signal using a branched equation framework [27] as described in previous publications from this cohort [28]. We primarily used the uniaxial acceleration signal to define MVPA in this analysis, as it has been suggested that mechanical loading has a stronger influence on bone than metabolic loading [29,30].

The acceleration signal was summarized as a fraction of time spent in 25 acceleration thresholds (m/s^2^) across the movement intensity continuum. Based on our previous research, which demonstrated MVPA, but not light PA or sedentary time, was associated with BMC, we used MVPA, categorized as acceleration >0.75 m/s^2^, as our PA exposure [4]. This threshold is based on previous research that assessed uniaxial acceleration during treadmill walking and running at different speeds [29,31]. The combined sensing data were used to characterize the proportion of children meeting the physical activity guidelines of 60 min MVPA per day, based on time spent >4 metabolic equivalents (METs), with 3.5 mL O_2_/min/kg used to define resting metabolic rate (1 MET) [32].

Non-wear time was classified as zero-acceleration lasting >90 min combined with non-physiological heart rate and diurnal imbalance in non-wear was minimized when summarizing the data to reduce bias and error as previously described [28,33]. Criteria for a valid PA measurement were ≥48 h of good-quality data with ≥32 h of weekday data and ≥16 h of weekend data as well as ≥12 h of morning, noon, afternoon and evening wear time to protect against bias from over-representation at specific times of day, and to optimize the diurnal bias minimization procedure [28,33].

### 2.6. Assessment of Free Leptin Index

Venous blood samples were taken from the children after 12 h of overnight fasting. The collected blood was centrifuged and then stored at −75 °C until analyses were conducted. Plasma leptin levels (ng/mL) were measured using a competitive radioimmunoassay (Multigamma 1261–001, PerkinElmer Wallac Oy, Turku, Finland) and plasma soluble leptin receptor concentration using an ELISA kit (Multicalc evaluation program PerkinElmer Wallac Oy, Turku, Finland). The within-day and between-day coefficients of variation were 2% and 4% for plasma leptin, and 7% and 5% for soluble leptin receptor, respectively. Free leptin index was calculated by dividing plasma leptin levels by the concentration of plasma soluble leptin receptor and multiplying by 100; free leptin index has been suggested to provide a more precise measure of the physiological actions of leptin [34].

### 2.7. Statistical Analysis

Analyses were performed with IBM SPSS Statistics for Mac software, Version 26.0 (IBM Corp., Armonk, NY, USA) and Stata/SE for Mac software, Version 16.1 (StataCorp LLC, College Station, TX, USA). Differences between included and excluded children were assessed using independent sample t-tests for age, body height and TBLH BMC, using Mann–Whitney U tests for TBLH fat mass and free leptin index, and using Fisher’s exact tests for weight status.

Age, body height, TBLH BMC and MVPA were normally distributed, whereas TBLH fat mass and free leptin index had a skewed distribution. Free leptin index had a curvilinear relationship with TBLH BMC, so a natural logarithmic transformation was used. We confirmed log-transformed free leptin index met assumptions of linearity with TBLH BMC. We calculated the mean and standard deviation (SD) for the total study sample, and for girls and boys separately, for normally distributed variables, and used independent samples t-tests to test for sex differences. We calculated the median and interquartile range (IQR) for the total sample, and for girls and boys separately, for skewed variables, and Mann–Whitney U tests were used to test for sex differences. Chi-squared tests were used to test for sex differences in categorical variables. Pearson correlation coefficients were utilized to identify associations between the independent, mediator and dependent variables. We tested whether free leptin index interacted with sex to influence TBLH BMC. As the free leptin index and sex interaction was significant with and without adjustment for relevant covariates, further analyses were stratified by sex.

**Figure 2:**
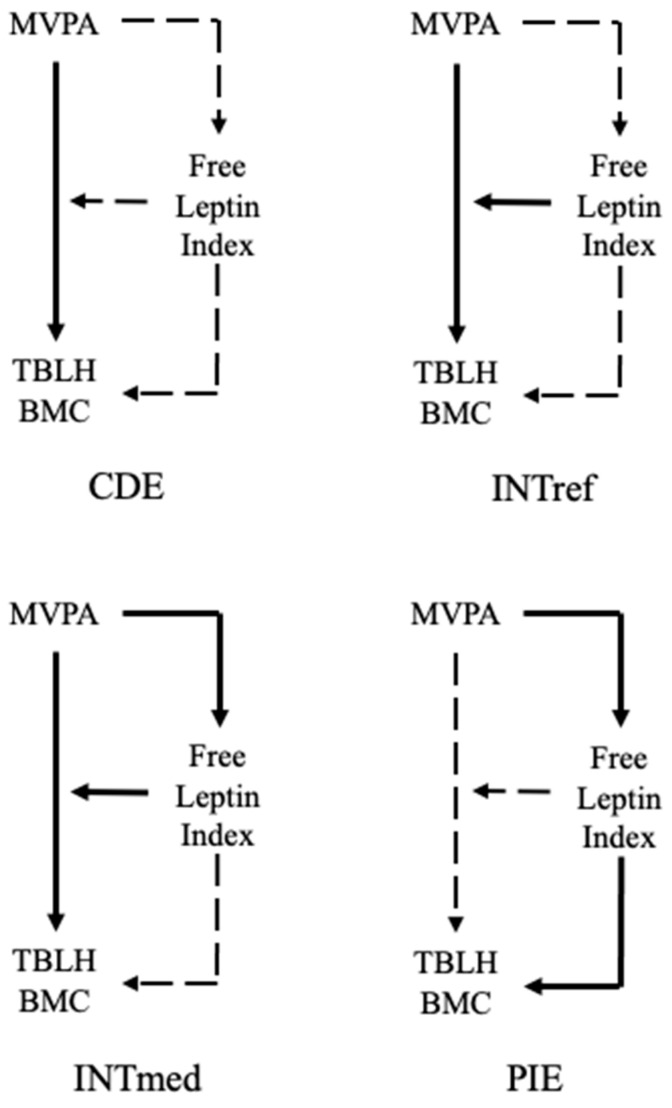
The components of the 4-way decomposition analysis as described by VanderWeele [13]. Controlled direct effect, CDE; reference interaction, INTref; mediated interaction, INTmed; pure indirect effect, PIE; moderate-to-vigorous physical activity, MVPA; total body less head bone mineral content; TBLH BMC. Solid lines show the pathway for the effect of interest while dashed lines show effects held constant.

We used the 4-way decomposition statistical approach developed by VanderWeele [13], and applied the Med4Way command in Stata/SE (Stata Statistical Software: Release 16. College Station, TX: StataCorp LLC.) [35], which allows the total relationship between PA and BMC to be separated into four components: CDE, INTref, INTmed, and PIE, shown in Figure 2. Two multivariable-adjusted regression models were fitted: the outcome model and the mediator model. The outcome model included MVPA, free leptin index, and an interaction term for the PA component and the free leptin index as independent variables, with TBLH BMC as the dependent variable. The mediator model included the PA component as the independent variable and free leptin index as the dependent variable. All models met the assumptions of normality, linearity and homoscedasticity in the residuals. Given the nature of mediation analyses, multicollinearity is expected. Due to high variance inflation factor (VIF) values, the MVPA and log-transformed free leptin index were mean-centered before being entered into analysis. With mean-centered variables, all VIF values were <5. Influential outliers were assessed using Cook’s distance, and cases with Cook’s distance substantially greater than the rest were considered outliers [36]. As outliers were identified in all models, analyses were run both including and excluding outliers to test the impact of outliers on the results. Results from the analysis excluding outliers were similar in terms of magnitude, direction and significance to those including outliers, so the results were presented with the inclusion of outliers.

All models were adjusted for chronological age, body height and TBLH fat mass. Estimates for the 4-way decomposition were computed fixing MVPA at the median and 75th percentile and free leptin index at the median. The alpha value for statistical significance was set as 0.05. Effect estimates (ß), 95% confidence intervals (CI) and *p*-values were reported.

## 3. Results

### 3.1. Characteristics of Children

Children excluded from this study did not differ from the included children in age, body height, TBLH BMC, or TBLH fat mass. In total, 20.5% of the excluded children were overweight or obese according to the IOTF definition, compared with 11.2% of the included children (*p* = 0.012) (Table S1). Of the included children, girls and boys did not differ in age or IOTF weight status (Table 1). Body height, TBLH BMC and MVPA (mins/day) were greater for boys than girls (*p* < 0.001), with a greater proportion of boys meeting the MVPA guidelines (*p* = 0.006), whilst TBLH fat mass and free leptin index were greater for girls (*p* < 0.001) (Table 1).

### 3.2. Associations between Mediator, Dependent and Independent Variables

Table 2 presents the partial correlations between MVPA, free leptin index and TBLH BMC by sex, adjusted for age, body height and TBLH fat mass. No significant associations were found between free leptin index and MVPA in boys or girls (*p* > 0.05). A weak, positive correlation was identified between MVPA and TBLH BMC in boys (*r* = 0.238, *p* = 0.001) and girls (*r* = 0.229, *p* = 0.001), respectively. Free leptin index presented a weak, negative association with TBLH BMC in both boys (*r* = −0.227, *p* = 0.001) and girls (*r* = −0.147, *p* = 0.043).

### 3.3. 4-Way Decomposition

**Figure 3:**
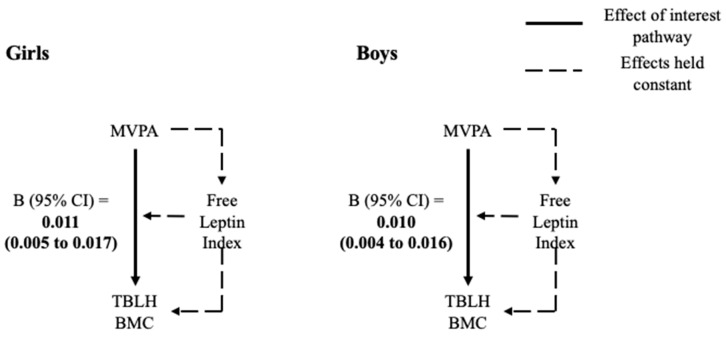
Controlled direct effect of MVPA on TBLH BMC from the 4-way decomposition model for girls and boys. Moderate-to-vigorous physical activity, MVPA; total body less head bone mineral content, TBLH BMC. Models adjusted for age, body height and TBLH fat mass.

A positive association was found between MVPA and TBLH BMC for both girls (β = 0.0004, 95% CI = 0.0002 to 0.0006, *p* = 0.001) and boys (β = 0.0003, 95% CI = 0.0001 to 0.0005, *p* = 0.001) in the outcome model (Table S2). Free leptin index was negatively associated with TBLH BMC for both girls (β = −0.0457, 95% CI = −0.0907 to −0.0006, *p* = 0.047) and boys (β = −0.0845, 95% CI = −0.1376 to −0.0314, *p* = 0.002). However, no interaction was found between MVPA and free leptin index in the outcome model for either girls or boys. No associations were found between MVPA and free leptin index in the mediator model (Table S2). In the four-way decomposition model of the association between MVPA and TBLH BMC mediated by free leptin index, the CDE was positive in girls (β = 0.011, 95% CI = 0.005 to 0.017, *p* < 0.001) and boys (β = 0.010, 95% CI = 0.004 to 0.016, *p* = 0.002) (Table 3 and Figure 3).

## 4. Discussion

Our study is the first to formally test the mediating and moderating role of the free leptin index using the 4-way decomposition method in the relationship between MVPA and TBLH BMC in a population sample of prepubertal children aged 6–9 years. Free leptin index did not mediate or moderate the association between MVPA with TBLH BMC in the 4-way decomposition model, indicating that MVPA influences BMC through pathways other than free leptin index, likely primarily through mechanical loading. Most of our sample were normal weight (89%), and the children excluded from the study were disproportionately overweight and obese; these findings may not apply to overweight and obese children, given that leptin is closely associated with adiposity [34]. However, as obesity is associated with earlier onset of puberty [37], and given that this study was in prepubertal children only, it follows that a greater proportion of overweight and obese children were in the excluded group compared to the included group. The median levels of plasma leptin observed in our sample (4.25 ng/mL in girls and 3.10 ng/mL in boys) were similar to those reported previously in non-obese girls and boys of the same age [38], which indicates the leptin levels in our sample (Table 1) are representative of children aged 6–9 years and of a healthy weight.

### 4.1. Free Leptin Index and Bone Mineral Content

Previous research into the associations between leptin and bone has been inconclusive, with evidence from in vitro and in vivo studies suggesting both the positive and negative mechanistic actions of free leptin index on BMC [39]. We observed a negative association between free leptin index and TBLH BMC in all models (Table 2). We have previously shown in this prepubertal sample that the positive association between free leptin index and bone mineral density was fully explained by the association between fat mass and bone mineral density [9].

Of previous studies that have taken fat mass into account, in children aged 7–8 years, plasma leptin was not associated with TBLH BMC [12]. Our sample were a similar age to those in Garnett et al.’s study [12], and we used DXA to assess TBLH BMC and fat mass, as did Garnett et al. [12], but the covariates we used in our models differed, which may explain these conflicting findings [12]. As leptin may act on bone through the hypothalamic–pituitary–gonadal and hypothalamic–pituitary–adrenal axes, the inclusion of sex steroids and insulin-like growth factor 1 by Garnett et al. [12] may have obscured the relationship between leptin and bone. In children and adolescents aged 5–16 years, free leptin index was inversely associated with bone turnover markers, indicating that greater levels of leptin could lead to decreased bone mass, supporting our findings [39]. This may explain a mechanism through which leptin negatively influences bone. However, it is unknown whether the association of leptin with bone turnover markers translates into changes in bone mass, which makes it difficult to draw direct comparisons between studies [39].

### 4.2. MVPA and Free Leptin Index

Taking fat mass into account, we did not observe associations between MVPA and free leptin index (Table 2). Our findings are in agreement with previous research in prepubertal girls and boys aged 8 years, which found that accelerometer-measured total PA and MVPA were not associated with leptin, taking body fat percentage into account [11]. However, in boys aged 12–14 years, accelerometer-measured MVPA was negatively related to changes in leptin over two years, after adjustment for BMI [5]. In female and male adolescents aged 12.5–17.5 years, average PA and vigorous PA, but not moderate PA, were negatively associated with leptin, independently of total body fat [40]. Similarly, in children and adolescents aged 8–18 years, pedometer-assessed step count was negatively associated with leptin in girls only, after controlling for the sum of skinfolds thickness [10].

Differences in pubertal status between study samples may explain the conflicting findings between studies. Studies in strictly prepubertal samples have not identified significant associations between PA and leptin [11], whereas those in pubertal or mixed samples have found negative associations between PA and leptin [5,10,40]. As leptin follows sex-specific and pubertal stage-specific patterns during puberty, it possible that the varying leptin levels between studies may explain the conflicting findings, whereby those studies with variation in pubertal status are likely to have greater variation in leptin, and may therefore be more likely to detect an association [41].

Differences in adiposity may also contribute to the conflicting findings, though this is difficult to assess as the proportions of overweight and obese in the sample populations were not characterized in previous studies [5,10], and pediatric experimental studies are limited to overweight and obese populations [42]. The only study to assess the associations between PA and leptin in a pediatric sample with mixed weight status found that the negative association between PA and leptin did not change when excluding overweight and obese adolescents (≈22%), indicating that weight status may not modify the effect of PA on leptin [40]. A meta-analysis of randomized controlled trials in children, adolescents and adults found that chronic exercise training (≥2 weeks) was associated with a decrease in plasma leptin regardless of changes in adiposity, though the effect was moderated by a decrease in body fat percentage; a greater decrease in body fat percentage was associated with a greater decrease in plasma leptin [8]. However, the authors highlight the lack of research in pediatric populations, and as the results did not change when excluding child and adolescent studies, it is unclear whether the association between PA and leptin in children is moderated by adiposity [8]. Even so, our data were fairly homogenous, with 89% of our sample being a normal weight (Table 1). It is possible that the studies that did find an association between PA and leptin had greater variations in fat mass and leptin, and were therefore better able to detect an effect [5,10,40]. Further research comparing the associations between PA and leptin in normal weight and overweight children would be valuable in exploring the potential moderating role of adiposity.

PA level seems unlikely to explain the conflicting findings between studies [5,11,40], as the average level of PA appeared consistent across all studies. In our sample, 79% met the PA guidelines of 60 min per day (Table 1), though it is well established that the proportion of children meeting the guidelines varies considerably based on the thresholds used [43]. The other study that did not observe an association between PA and leptin observed an average of 56 min/day MVPA [11], and those that did find a relationship between PA and leptin observed averages of 59 min/day MVPA [40] and 65 min/day MVPA [5]. Although it is difficult to draw direct comparisons between studies, it seems unlikely that differences in PA level are responsible for the conflicting findings.

### 4.3. MVPA, Free Leptin Index and Bone Mineral Content

We did not find support for INTref, INTmed or PIE through free leptin index in the MVPA with bone relationship (Table 3). MVPA had a positive CDE with TBLH BMC in girls and boys. To our knowledge, only one previous pediatric study has considered PA, leptin and BMC [5]. In boys aged 12–14 years, MVPA was negatively associated with leptin, and leptin was negatively associated with BMC, controlling for BMI [5]. However, a formal mediation analysis was not conducted by Vaitkeviciute et al. [5], so drawing direct comparisons is challenging. As previously discussed, we did not observe associations between PA and free leptin index, yet the prior reasons outlined likely explain why our findings differ to those of Vaitkeviciute et al. [5]. Furthermore, we controlled for fat mass in our analysis, whereas Vaitkeviciute et al. [5] controlled for BMI. Although controlling for BMI is likely to account for some of the same variance as fat mass, research indicates that BMI explains 57–73% variance in DXA-measured fat mass [44]. As fat mass is an important confounder when investigating leptin, this could explain why our findings differ from those of Vaitkeviciute et al. [5].

We have previously demonstrated the positive association between MVPA and TBLH BMC in prepubertal children [4], and this has been consistently demonstrated in previous pediatric studies [30,45,46], indicating MVPA is a positive predictor of BMC. The positive CDE of MVPA with TBLH BMC (Table 3) that we observed indicates that in prepubertal children of a healthy weight, MVPA positively influences TBLH BMC through pathways not related to free leptin index, likely through directly increasing loads on bones, and by increasing lean mass, which increases forces on bones [45]. We have previously discussed the clinical relevance of the associations between MVPA and bone mass in prepubertal children elsewhere [4].

### 4.4. Limitations

Our findings should be considered in the context of the limitations of our study. The assessment of bone mass is challenging in growing children. We used TBLH BMC, as recommended by the International Society for Clinical Densitometry, adjusted for age and body height, which are components of the height z-score [47]. However, true volumetric bone mineral density cannot be determined from DXA, and associations with bone geometry cannot be assessed [17,48]. Future studies using peripheral quantitative computed tomography would be valuable in extending the research to assess the geometric properties of bone [48]. Although we controlled for fat mass, a key confounder when investigating leptin, in addition to age and body height, residual confounding remains a potential limitation in all observational studies. Evidence for causal relationships cannot be provided by cross-sectional studies, and there remains a possibility of bidirectional relationships.

## 5. Conclusions

Our study indicates that free leptin index is negatively associated with TBLH BMC, but not associated with MVPA, and that MVPA positively influences TBLH BMC through pathways not related to free leptin index in normal-weight prepubertal children aged 6–9 years old. The relationships between MVPA and leptin may be influenced by other factors, such as pubertal status and adiposity, so it is unknown whether these observations extend to overweight and obese children at different stages of puberty.

## Figures and Tables

**Table 1:** Descriptive characteristics of included children.

	Total	Girls	Boys	*p* Value for Sex Difference
	*n* = 401	*n* = 194	*n* = 207	
	Mean (SD)/ Median (IQR)	Mean (SD)/ Median (IQR)	Mean (SD)/ Median (IQR)	
Age (years)	7.65 (0.39)	7.61 (0.37)	7.68 (0.40)	0.105
Body height (cm)	128.65 (5.63)	127.46 (5.56)	129.77 (5.49)	**<0.001**
IOTF definition				
% (cases) normal weight	88.8 (356)	88.7 (172)	88.9 (184)	0.970
% (cases) overweight	8.0 (32)	8.2 (16)	7.7 (16)	
% (cases) obese	3.2 (13)	3.1 (6)	3.4 (7)	
MVPA recommendations (>4 METs)				
% (cases) ≥ 60 min/day	78.6 (315)	72.7 (141)	84.1 (174)	**0.006**
% (cases) < 60 min/day	21.4 (86)	27.3 (53)	15.9 (33)	
TBLH BMC (g)	670.78 (132.51)	645.47 (126.12)	694.49 (134.26)	**<0.001**
TBLH fat mass (kg)	4.34 (2.96 to 6.19)	4.73 (3.55 to 6.67)	3.79 (2.42 to 5.83)	**<0.001**
Plasma leptin (ng/mL)	3.70 (2.70 to 5.75)	4.25 (3.20 to 6.60)	3.10 (2.40 to 4.70)	**<0.001**
Free leptin index	8.82 (5.84 to 16.14)	10.63 (7.35 to 19.22)	6.87 (5.11 to 12.82)	**<0.001**
MVPA (mins/day spent > 0.75 m/s^2^)	130.08 (42.47)	121.30 (36.82)	138.31 (45.73)	**<0.001**

International Obesity Task Force, IOTF; total body less head, TBLH; bone mineral content, BMC; moderate-to-vigorous physical activity, MVPA. Values in bold indicate significant *p*-values (*p* < 0.05).

**Table 2:** Partial correlations of independent, mediator and dependent variables according to sex.

	Free Leptin Index		TBLH BMC (g)	
	*r/p*-Value	Result	*r/p*-Value	Result
**MVPA (> 4 METs)**				
Girls	0.044/0.542		0.229/**0.001**	
		No relationship between free leptin index and MVPA, controlled for age, body height and TBLH fat mass		Weak positive relationship between BMC and MVPA, controlled for age, body height and TBLH fat mass
Boys	−0.094/0.182		0.238/**0.001**	
		
All	−0.055/0.277		0.261/ **< 0.001**	
**Free Leptin Index**				
Girls	–		−0.147/**0.043**	
				Weak negative relationship between BMC and free leptin index, controlled for age, body height and TBLH fat mass
Boys	–		−0.227/**0.001**	
		
All	–		−0.227/ **< 0.001**	

All models adjusted for age, body height, and TBLH fat mass. Moderate-to-vigorous physical activity, MVPA; total body less head fat mass, TBLH FM. Values in bold indicate significant *p*-values (*p* < 0.05).

**Table 3:** Four-way decomposition of the association between MVPA^α^ and TBLH BMC.

	Girls (*n* = 194)		Boys (*n* = 207)	
	β (95% CI)	*p*-value	β (95% CI)	*p*-Value
**Free Leptin Index**				
TE	**0.0110 (0.0048 to 0.0171)**	**<0.001**	**0.0104 (0.0043 to 0.0164)**	**0.001**
CDE	**0.0113 (0.0052 to 0.0173)**	**<0.001**	**0.0095 (0.0035 to 0.0155)**	**0.002**
INTref	0.0000 (−0.0003 to 0.0004)	0.811	0.0000 (−0.0004 to 0.0004)	0.938
INTmed	−0.0000 (−0.0004 to 0.0002)	0.570	−0.0000 (−0.0002 to 0.0002)	0.938
PIE	−0.0003 (−0.0011 to 0.0006)	0.556	0.0008 (−0.0005 to 0.0022)	0.214

All models adjusted for age, body height, and TBLH fat mass. Total body less head, TBLH; total effect, TE; controlled direct effect, CDE; reference interaction, INTref; mediated interaction, INTmed; pure indirect effect, PIE. ^α^Free leptin index was log-transformed. ^α^MVPA was mean-centered. The values at which the 4-way decomposition was computed are presented in Table S3. Values in bold indicate significant *p*-values (*p* < 0.05).

## Data Availability

The datasets analyzed during the current study are available from the corresponding author on reasonable request.

## Supplementary Materials

The following are available online at https://www.mdpi.com/article/10.3390/ijerph18105365/s1, Table S1. Differences in participant characteristics between included and excluded children; Table S2. Associations between MVPA, free leptin index and total body less head bone mineral content (outcome model) and associations between free leptin index and MVPA (mediator model); Table S3. Fixed values used in Med4Way analysis.
