# Supplementary material for: The Positive Relationship between Moderate-to-Vigorous Physical Activity and Bone Mineral Content Is Not Mediated by Free Leptin Index in Prepubertal Children: The PANIC Study
Source: Int J Environ Res Public Health. 2021 May 18;18(10):5365. doi: 10.3390/ijerph18105365 (PMC8157575; doi:10.3390/ijerph18105365)
Supplement: Supplementary file 1 [file ijerph-18-05365-s001.zip › ijerph-1212057-supplementary.pdf]

International Journal of
Environmental Research and Public Health

MDPI

Table S1. Differences in participant characteristics between included and excluded children.

	**Included children (*n* = 401)**	**Excluded children (*n* = 83 to 103)**	***p* value for group difference**
	Mean (SD) / Median (IQR)	Mean (SD) / Median (IQR)	
Age (years)	7.6 (0.4)	7.6 (0.4)	0.182
Stature (cm)	128.7 (5.6)	129.3 (5.9)	0.312
IOTF Definition			
% (cases) normal weight	88.8 (356)	79.6 (82)	**0.025**
% (cases) overweight	8.0 (32)	11.7 (12)	
% (cases) obese	3.2 (13)	8.7 (9)	
TBLH BMC (g)	0.67 (0.13)	0.70 (0.17)	0.051
TBLH FM (kg)	4.34 (2.96 to 6.19)	4.59 (2.84 to 8.69)	0.217
Free Leptin Index	8.82 (5.84 to 16.14)	10.02 (5.88 to 22.19)	0.194

International obesity task force, IOTF; total body less head, TBLH; bone mineral content, BMC; fat mass, FM.; Values in bold indicate significant p-values (< 0.05). Number of children (n) varies for different variables; n = 504, 401 included and 103 excluded: age, stature, IOTF definition; n = 493, 401 included and 92 excluded: TBLH BMC, TBLH FM; n = 484, 401 included and 83 excluded: Free Leptin Index.

Table S2. Associations between MVPA, free leptin index and total body less head bone mineral content (outcome model) and associations between free leptin index and MVPA (mediator model).

	**Girls (*n* = 194)**		**Boys (*n* = 207)**	
	**β (95% CI)**	***p*-value**	**β (95% CI)**	***p*-value**
**Outcome Model**				
MVPA$^{\alpha}$	0.0004 (0.0002 to 0.0006)	**0.001**	0.0003 (0.0001 to 0.0005)	**0.001**
Free Leptin Index$^{\alpha}$	-0.0457 (-0.0907 to -0.0006)	**0.047**	-0.0845 (-0.1376 to -0.0314)	**0.002**
MVPA$^{\alpha}$ X Free Leptin Index$^{\alpha}$	-0.00051 (-0.0013 to 0.0002)	0.155	0.0000 (-0.0005 to 0.0006)	0.938
**Mediator Model**				
Free Leptin Index$^{\alpha}$	0.0002 (-0.0005 to 0.0009)	0.536	-0.0004 (-0.0009 to 0.0002)	0.175

All models adjusted for age, stature, and TBLH fat mass. Total body less head, TBLH; Moderate-to-vigorous physical activity, MVPA. $^{\alpha}$ Free Leptin Index was log-transformed. $^{\alpha}$ MVPA was mean-centred. Values in bold indicate significant p-values ($p < 0.05$).

Table S3. Fixed values used in Med4Way analysis.

	**Girls**	**Boys**
MVPA reference value (median value)	118.3406	136.5738
MVPA actual value (75th percentile)	145.2338	164.3532
Log transformed Free Leptin Index (median value)	1.0265	0.8370
TBLH FM (median value)	4.7250	3.7890
Stature (median value)	128.000	130.300
Age (median value)	7.6350	7.6400

Moderate-to-vigorous physical activity, MVPA; total body less head fat mass, TBLH FM.
